# Mouse Visual Cortex Is Modulated by Distance Traveled and by Theta Oscillations

**DOI:** 10.1016/j.cub.2020.07.006

**Published:** 2020-10-05

**Authors:** Julien Fournier, Aman B. Saleem, E. Mika Diamanti, Miles J. Wells, Kenneth D. Harris, Matteo Carandini

**Affiliations:** 1 UCL Institute of Ophthalmology, University College London, London EC1V 9EL, UK; 2 Neuroscience Paris-Seine – Institut de biologie Paris-Seine, Sorbonne Universités, INSERM, CNRS, Paris, France; 3 Laboratoire des systèmes perceptifs, DEC, ENS, PSL University, CNRS, 75005 Paris, France; 4 UCL Institute of Behavioural Neuroscience, Department of Experimental Psychology, University College London, London WC1H 0AP, UK; 5 CoMPLEX, Department of Computer Science, University College London, London WC1E 7JG, UK; 6 UCL Queen Square Institute of Neurology, University College London, London WC1N 3BG, UK

**Keywords:** primary visual cortex, hippocampus, navigation, distance coding, theta oscillation, virtual reality

## Abstract

The visual responses of neurons in the primary visual cortex (V1) are influenced by the animal’s position in the environment [1, 2, 3, 4, 5]. V1 responses encode positions that co-fluctuate with those encoded by place cells in hippocampal area CA1 [2, 5]. This correlation might reflect a common influence of non-visual spatial signals on both areas. Place cells in CA1, indeed, do not rely only on vision; their place preference depends on the physical distance traveled [6, 7, 8, 9, 10, 11] and on the phase of the 6–9 Hz theta oscillation [12, 13]. Are V1 responses similarly influenced by these non-visual factors? We recorded V1 and CA1 neurons simultaneously while mice performed a spatial task in a virtual corridor by running on a wheel and licking at a reward location. By changing the gain that couples the wheel movement to the virtual environment, we found that ∼20% of V1 neurons were influenced by the physical distance traveled, as were ∼40% of CA1 place cells. Moreover, the firing rate of ∼24% of V1 neurons was modulated by the phase of theta oscillations recorded in CA1 and the response profiles of ∼7% of V1 neurons shifted spatially across the theta cycle, analogous to the phase precession observed in ∼37% of CA1 place cells. The influence of theta oscillations on V1 responses was more prominent in putative layer 6. These results reveal that, in a familiar environment, sensory processing in V1 is modulated by the key non-visual signals that influence spatial coding in the hippocampus.

## Highlights

• We compared responses of mouse V1 and CA1 during navigation in virtual reality
• Similar to CA1 neurons, V1 neurons are influenced by physical distance traveled
• V1 responses are modulated by theta oscillations recorded in hippocampus
• Positions encoded by V1 shift spatially with the phase of the theta oscillation

## Graphical Abstract

**Figure:**
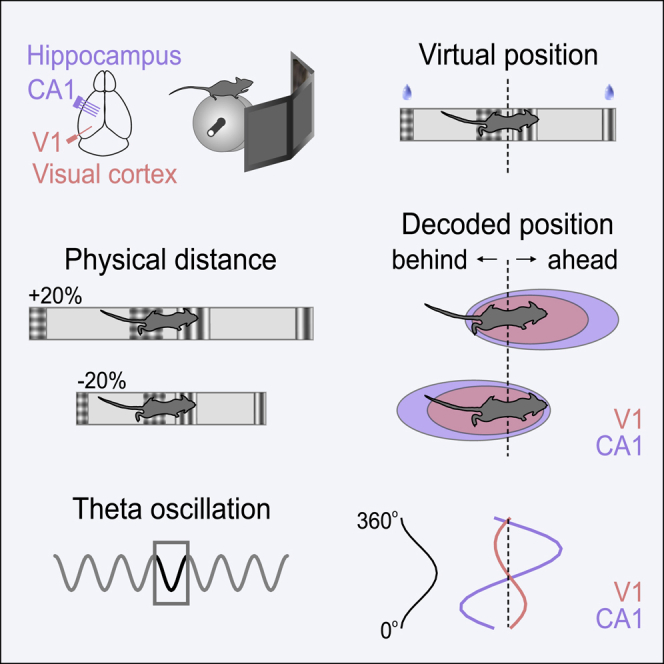

## Teaser

Fournier et al. compare responses of hippocampus and visual cortex in mice navigating a virtual reality. They find that, similar to hippocampus, activity in visual cortex is influenced by the physical distance traveled in an environment. In addition, theta oscillations modulate the activity of visual cortical neurons and their position coding.

## Results

**Figure 1:**
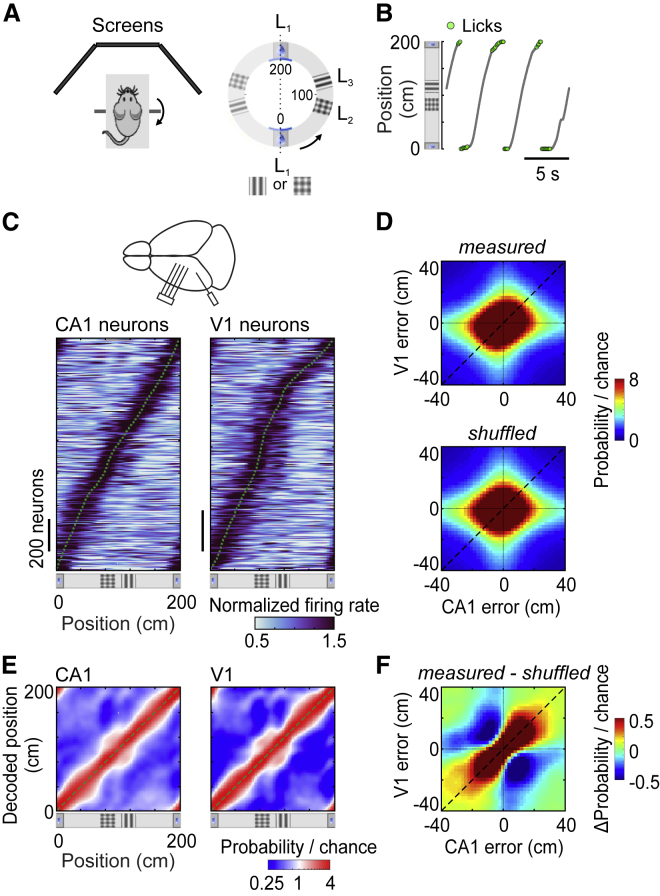
Coherent Spatial Representations in CA1 and V1 in a Visually Unambiguous Environment (A) Schematic of the setup: running wheel surrounded by three screens (left) and virtual corridor with landmarks (L1, L2, and L3, right). See also Figures S1. (B) Example trajectories, showing the locations of licks (green dots). (C) Top: schematic of recordings with silicon probes in hippocampus (CA1) and visual cortex (V1). Shown on the bottom are responses of neurons in CA1 (left, n = 1,431) and V1 (right, n = 1,127) that significantly changed firing rate along the corridor (p < 0.01, permutation test) Neurons are ordered by the position of maximal firing (dotted green curve). Each response was normalized by the mean firing rate across positions. (D) Joint distribution of V1 and CA1 decoding errors, averaged across spatial positions and sessions (n = 27). Shown at the top is the joint distribution obtained from the measured responses. Shown on the bottom is the joint distribution obtained after shuffling time points within position and speed bins. (E) Distribution of positions decoded from CA1 (left) or V1 (right), as a function of the animal’s position, averaged across all trials. (F) Difference between joint distributions of V1 and CA1 errors obtained from measured responses and shuffled control. See also Figure S2.

To test whether responses in visual cortex are modulated by non-visual factors that affect spatial coding in hippocampus, we recorded from both regions simultaneously while mice performed a spatial task in virtual reality (Figures 1A–1C). Head-fixed mice ran on a wheel to explore a virtual corridor defined by landmarks in three positions (L1–L3) (Figure 1A). The corridor was semicircular and repeated into a full circle without interruption between trials. We trained mice to lick near landmark L1 for a water reward. To encourage mice to use strategies beyond the discrimination of visual textures, the texture at landmark L1 alternated between the textures shown at L2 and L3 (a plaid and a grating, Figure 1A). After ∼6–8 weeks, mice licked exclusively when approaching the reward zone in more than 80% of the trials (Figure 1B). We then used silicon probes to record from CA1 and V1 simultaneously (Figure 1C). Neurons in both V1 and CA1 responded similarly regardless of the texture shown in the reward position (grating or plaid), so we pooled all trials together (Figures S1A–S1C). We focused on neurons that significantly changed their firing rate across positions in the corridor (1,431 CA1 neurons from 54 recording sessions, excluding putative interneurons; 1,127 V1 neurons from 35 recording sessions; p < 0.01). Positions along the corridor were visually distinct, so these responses could be due to visual cues, spatial positions or both.

As expected [5], neural populations in both CA1 and V1 had response profiles that tiled the corridor, providing a coherent encoding of spatial position (Figures 1C–1F). Tiling in CA1 was more uniform than in V1, where more cells responded near visual landmarks. The activity of either region could be used to decode the animal’s position (Figure S2A). On average, both CA1 and V1 neural responses reflected the position of the animal (Figure 1E). As previously shown [5], occasional errors in the positions encoded by V1 and CA1 were significantly correlated (Figures 1D and 1F). This correlation could not be explained by fluctuations in other aspects of the animal’s behavior (Figures S2B–S2F).

### V1 Neurons Are Influenced by the Distance Traveled in the Environment

The correlation between V1 and CA1 errors could reflect a common effect of non-visual factors in both areas. A key non-visual factor affecting spatial coding in CA1 is idiothetic information: the distance traveled in the environment [6, 7, 8, 9, 10, 11, 14]. Might this factor similarly affect responses in V1?

**Figure 2:**
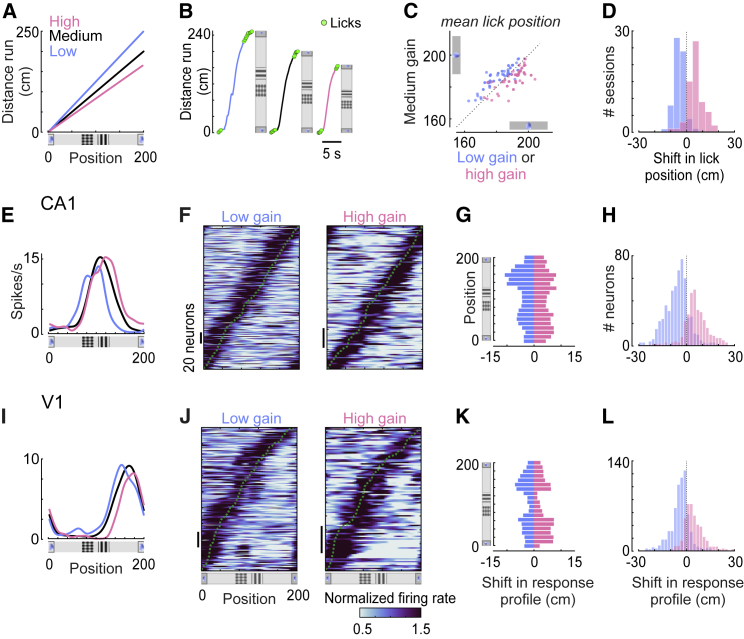
V1 and CA1 Neurons Are Modulated by the Distance Traveled in the Environment (A) Physical distance traveled from landmark L1 on trials with low, medium, and high gain. (B) Examples of single-trial trajectories for trials with low (blue), medium (black), and high gain (pink), showing the position of licks (green dots). (C) Visual position of licks on trials with low (blue) or high (pink) gain compared to medium gain. Each point is the average for a session. (D) Distribution of the shift in mean lick position across sessions in trials with low and high gain. (E) Response of a CA1 neuron on trials with low, medium, and high gain. (F) Response of CA1 neurons that showed a significant spatial shift of their response (in any direction) on trials with low gain (left; 42.4% of 559 neurons; p < 0.05, permutation test) or high gain (right; 40.4% of 329 neurons; p < 0.05). Neurons are ordered according to the position of maximal firing on trials with medium gain (dotted curve). (G) Median spatial shifts at low (blue) or high (pink) gain across all CA1 neurons as a function of the position of their peak response. Spatial shifts were measured in relation to the response at medium gain. (H) Distribution of spatial shifts in CA1 responses at low (blue) or high (pink) gain. (I) Response profile of a V1 neuron on trials with low, medium, and high gain. (J) Same as (F) for neurons in V1 (low gain; 24.4% of 667 neurons; high gain; 21.1% of 446 neurons, p < 0.05, permutation test). (K–L) Same as (G)–(H) for neurons in V1. See also Figure S3.

To investigate the effect of the distance traveled, we changed the gain of the wheel by ±20% in a fraction of trials (Figures 2A and 2B). In those trials (36% ± 13%, SD), mice had to run a longer or shorter distance than usual to reach any visual position. This manipulation had a small but consistent effect (Figures 2C and 2D): mice tended to lick 2.4 ± 0.2 cm earlier when the physical distance was longer (low gain, SE, p < 10^−6^, signed-rank test), and 2.5 ± 0.3 cm later when the physical distance was shorter (high gain, SE, p < 10^−6^, signed-rank test).

The physical distance traveled thus appeared to influence the animal’s own estimate of position; we next asked whether it also influenced the activity of CA1 and V1 neurons. To compare responses across gain conditions, we focused on neurons whose firing rate depended significantly on position, at both medium- and low-gain (559 out of 1,431 CA1 neurons; 667 out of 1,127 V1 neurons; p < 0.01) or both medium- and high-gain (329 out of 1,431 CA1 neurons; 446 out of 1,127 V1 neurons; p < 0.01).

Changes in gain affected spatial coding not only in CA1 [6, 7, 8, 9, 10, 11] but also in V1 (Figures 2E–2L). Changing gain did not affect mean firing rates (Figures S3A and S3B) but moved the position where neurons responded in relation to visual cues. As expected [6, 7, 8, 9, 10, 11], CA1 place cells tended to fire at earlier visual positions when the distance run was longer (low gain, 42.4% of 559 neurons, p < 0.05) and at later visual positions when the distance was shorter (high gain, 40.4% of 329 neurons, p < 0.05) (Figures 2E–2H). Many V1 neurons behaved similarly: even though the landmarks were encountered at the same virtual positions, the neurons fired earlier on the virtual track when the physical distance traveled was longer (low gain; 24.4% of 667 neurons, p < 0.05) and later when the distance was shorter (high gain; 21.1% of 446 neurons, p < 0.05) (Figures 2I–2L). For V1 neurons whose response was significantly influenced by distance traveled, this spatial shift accounted for >10% of the variance of the response profiles at low (10.5%) and high (13.3%) gain; for CA1 neurons, it accounted for ∼19% of the variance (low gain: 18.9%; high gain: 18.6%) (Figures S3E–S3I).

The shift in response profiles tended to be larger for neurons that fired maximally between landmarks (Figure 2G and 2K). This effect was significant in V1 at low and high gain (p < 0.002 and p < 0.016) and in CA1 at high gain (low gain, p < 0.08; high gain, p < 0.046), suggesting that in both regions, signals related to the physical distance traveled are corrected by salient visual landmarks. Shifts in responses of V1 neurons were independent of recording depth (Figures S3C and S3D), or cell type, i.e., putative interneurons and pyramidal cells (low gain: p = 0.90; high gain: p = 0.94, rank-sum test). These shifts could not be explained by changes in running speed, licking, or eye position (Figures S3J and S3K) or by interactions between the latency of visual responses in V1 and the speed of the virtual environment (Figures S1L–S1O).

**Figure 3:**
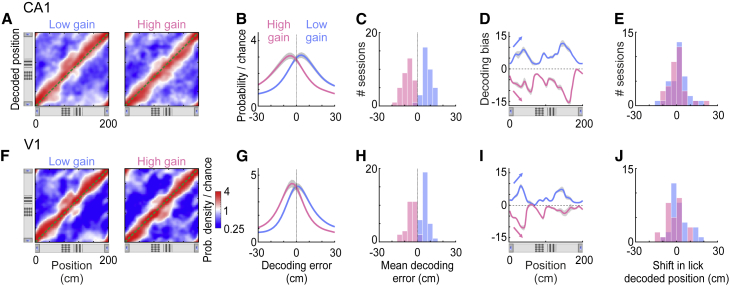
Positions Encoded in V1 and CA1 Populations Are Influenced by Distance Traveled and Consistent with Behavior (A) Distribution of positions decoded from CA1, as a function of the animal’s actual position, averaged across trials with low (left) or high (right) gain. Gray band shows ± SEM. (B) Distribution of errors in the position decoded from CA1 at low (blue) and high (pink) gain, averaged across sessions. (C) Distribution of mean decoding errors across CA1 recording sessions at low (blue) and high (pink) gain. (D) Decoding bias (i.e., most likely CA1 decoding error) at low (blue) and high (pink) gain, calculated across positions. Arrows indicate how the bias would accumulate if the decoded position depended only on distance traveled. Gray band shows ± SEM. (E) Distributions of the shift in mean lick position across sessions, using the positions decoded from CA1 at low (blue) and high (pink) gain, showing no significant differences. (F)–(J) Same as (A)–(E) for V1.

The physical distance traveled also influenced positions encoded at the level of populations (Figure 3). Both CA1 and V1 tended to encode a position ahead of the animal when the distance traveled was longer (low gain) and behind the animal when the distance was shorter (high gain) (Figures 3A–3C and 3F–3H). As with medium gain, trial-by-trial fluctuations in the positions decoded from both regions were correlated at low and high gain (Figure S2D). Similar to what we observed in individual neurons (Figures 2G and 2K), positions decoded from V1 or CA1 deviated more from the actual position when the animal was between landmarks (Figures 3D and 3I). This influence of distance traveled on decoded positions was observed regardless of speed and eye position (Figures S3P–S3R). Navigational cues thus modulated spatial coding in V1 and CA1 in a consistent way; distance estimates were corrected by salient visual landmarks.

The errors in positions decoded from V1 or CA1 were consistent with the animal’s decision to lick (Figures 3E and 3J). As previously shown [5], when mice made mistakes and licked too early or too late, the trajectories decoded from both V1 and CA1 populations traveled to the reward zone too early or too late (Figures S2G–S2L). This correlation persisted on correct trials at low and high gain: mice tended to lick earlier on low-gain trials and later on high-gain trials (Figure 2D). However, when licks were expressed as a function of positions decoded from V1 or CA1, there was no significant shift in their positions at low or high gain (Figures 3E and 3J) (CA1: p_low_ = 0.12, p_high_ = 0.90; V1: p_low_ = 0.18, p_high_ = 0.25, signed-rank test). Therefore, whether the physical distance traveled was long or short, the mouse on average licked in the same location in relation to positions encoded by V1 or CA1.

### V1 Neurons Are Influenced by the Theta Oscillation Recorded in Hippocampus

**Figure 4:**
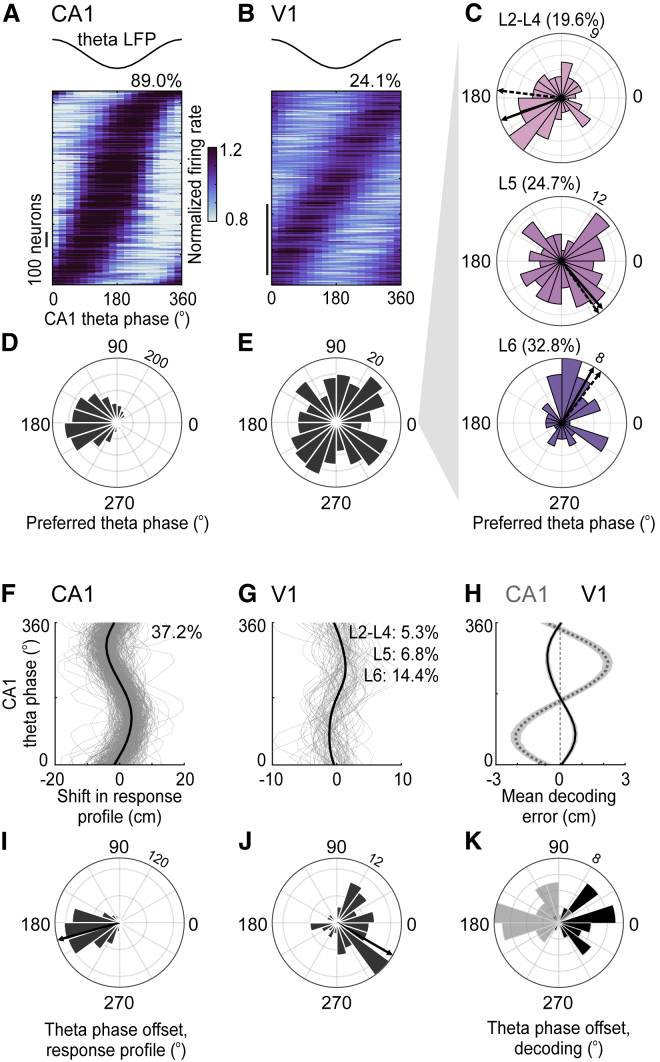
V1 Neurons Are Influenced by the Theta Oscillation Recorded in the Hippocampus (A) Firing rate of CA1 neurons as a function of the phase of theta oscillations recorded in hippocampus (top). Each neuron’s firing rate was normalized by its mean rate across phases. Neurons were ordered according to the theta phase of maximal firing. Only neurons with a significant modulation of firing rate across theta phase are represented (n = 1,274 out of 1,431; p < 0.05, permutation test). (B) Same as (A), for V1 neurons in relation to the theta oscillation measured in CA1 (n = 272 out of 1,127; p < 0.05, permutation test). See also Figure S4. (C) Distribution of the theta phase of maximal firing for V1 neurons recorded at different depths (L2–L4: 250–500 μm; L5: 500–700 μm; L6: 700–950 μm). Arrow indicates median of the preferred theta phase measured across neurons (solid) or across sessions (dashed). (D) Distribution of the theta phase of maximal firing for the CA1 neurons in (A). (E) Same for the V1 neurons in (B). (F) Spatial shift in response profiles across theta phases for CA1 neurons with a significant shift (gray curves: 37.2%, n = 1,431 neurons; p < 0.05, permutation test; black: average). (G) Same as (F) for V1 neurons with a significant spatial shift across theta phases (7.5%, n = 1,127 neurons; L6: 14.4% ± 2.9% (MAD), n = 188 neurons; L5: 6.8% ± 1.1%, n = 543 neurons; L2–L4: 5.3% ± 1.4%, n = 396 neurons, p < 0.05, permutation test). Note the smaller scale in (G) in relation to (F). See also Figure S4. (H) Mean error in population decoding from CA1 (gray) and V1 (black) as a function of the phase of the CA1 theta oscillation, averaged across sessions. Gray band shows ± SEM (CA1, n = 40 sessions; V1, n = 32 sessions). (I) Distribution of the theta phase offset (phase where the spatial shift went from positive to negative), for CA1 neurons with a significant spatial shift across theta phase. Arrow indicates median phase offset across all neurons. (J) Same as (I) for V1 neurons. (K) Distribution of the theta phase offset (phase where the decoding error went from behind to ahead) measured from the mean decoding error for each session, when decoding from CA1 (gray*)* and V1 (black).

As expected, CA1 neurons were strongly entrained by the 6–9 Hz theta oscillation [12, 13, 15] (Figures 4A and 4D). This oscillation modulated the firing rate of 89.0% of the CA1 place cells (n = 1,431 neurons, p < 0.05) (Figures 4A and S4A); firing rates were highest around 180° theta phase (p < 10^−180^, Rayleigh test) (Figure 4D).

We observed a similar entrainment in V1 (Figure 4B). The theta oscillation recorded in the hippocampus significantly modulated the firing rate of 24.1% of V1 neurons (n = 1,127 with p < 0.05; p < 10^−20^, Fisher’s combined probability test) (Figure S4A). The modulation of firing rates by theta phase accounted for 7.1% ± 3.9% (median absolute deviation [MAD]) of the variance of V1 response profiles across theta phases and positions; for CA1 neurons, it accounted for 20.7% ± 11.2% (Figures S4F–S4H). V1 neurons modulated by theta oscillations responded throughout the corridor (Figure S4B). These results extend similar observations in somatosensory and prefrontal cortices [16, 17, 18, 19]. As in those regions [16], in V1 the theta oscillation modulated narrow-spiking putative interneurons more than putative pyramidal neurons (Int.: 35.5%; Pyr.: 21.0%).

The modulation of V1 firing rates by theta oscillations depended on cortical layer (Figures 4C and 4E). V1 neurons were more entrained by theta oscillations in deep layers (L6: 32.8 ± 5.3% MAD, n = 188) than in intermediate (L5: 24.7% ± 2.5% n = 543, p = 0.056) or upper layers (L2–L4: 19.6% ± 2.4%; n = 396, p < 0.002) (Figures S4C and S4D). Moreover, their preferred theta phase depended on cortical depth (Figure 4C) (L6 versus L5: p = 0.002; L6 versus L2–L4: p = 0.04, non-parametric test for equal medians): deep neurons (putative layer 6) fired preferentially at ∼80° theta phase (p = 0.015, Rayleigh test), superficial neurons (putative layers 2–4) preferred ∼210° (p = 0.025, Rayleigh test), and intermediate neurons (putative layer 5) had intermediate preferences (p = 0.15, Rayleigh test). This change in preferred theta phase across V1 layers was significant across sessions (Figure S4E) (L6 versus L5: p = 0.008; L6 versus L2–L4: p = 0.002).

In the hippocampus, the theta oscillation also modulates spatial selectivity: place cells fire ahead of their preferred position at late phases of the theta cycle (180°–360°) and behind it at early phases (0° to 180°) [12, 13] (Figures 4F and 4I). This theta phase precession was significant in 37.2% of CA1 neurons (Figures 4F and S4K, p < 0.05) with a typical phase offset near 180° theta phase (Figure 4I). Over the CA1 population, the amplitude and phase offset of this theta phase precession were similar across gain conditions (medium, low, or high).

Intriguingly, the hippocampal theta oscillation also affected positions decoded from V1 (Figures 4H and 4K). As expected, positions decoded from CA1 populations shifted ahead of the animal at late theta phases and behind it at early theta phases (Figures 4H and 4K) (p < 0.002, permutation test). A similar shift was seen when decoding from V1 (Figures 4H and 4K) (p < 0.002, permutation test). The shift was, however, opposite to the one measured in CA1: V1 neurons indicated a position behind the animal at late theta phases and ahead at early theta phases (Figures 4H and 4K). This theta-dependent shift in decoded positions was significant in 34% of the sessions in V1 (11 out of 32; Fisher’s combined probability test, p < 10^−9^); in CA1, it was significant in 88% of the sessions (35 out of 40; p < 10^−48^). Moreover, the amplitude of the modulation of decoding errors by theta phase was correlated between V1 and CA1 on a cycle-by-cycle basis (Figure S4T).

The shift of spatial selectivity with theta phase could also be seen in some individual V1 neurons, particularly in deep layers (Figure 4G). Phase precession was significant in a small fraction of V1 neurons (7.5%; p < 0.05, n = 1,127) (Figures 4G and S4L–S4N) and was more prominent in putative layer 6 (L6: 14.4% ± 2.9% MAD; L5: 6.8% ± 1.1%, p = 0.006; L2–L4: 5.3% ± 1.4%, p = 0.002) (Figure S4O). The shift in V1 responses across theta phases was opposite to that observed in CA1 (theta phase offset between 270° and 360°) (Figure 4J), regardless of cortical depth (Figures S4P–S4Q). It was also smaller (Figure S4R) and more symmetrical in V1 neurons than in CA1 neurons (Figure S4S). This theta-dependent shift accounted for 1.0% ± 0.4% (MAD) of the variance of V1 response profiles; for CA1, it accounted for 2.9% ± 1.2% (Figures S4F, S4I, and S4J).

We thus found that positions encoded by V1 are modulated by the theta oscillation recorded in hippocampus. Albeit smaller, this modulation echoes the theta phase precession observed in CA1 with a systematic phase offset.

## Discussion

We have examined two non-visual factors that shape spatial coding in the hippocampus: the physical distance traveled [6, 7, 8, 9, 10, 20] and the hippocampal theta oscillation [12, 13]. We found that both of these factors influence the activity of neurons in the primary visual cortex (V1).

We created a virtual environment where we could probe the effect of distance traveled by changing the gain that relates the wheel to the virtual corridor [6]. Doing so revealed that V1 is influenced by the physical distance traveled: many V1 neurons shifted their responses away from the position of visual cues and toward the physical distance at which the animal would have otherwise encountered those cues. This shift was smaller around salient visual landmarks, suggesting that visual cues work as reference points where distance estimates are reset or (perhaps equivalently) that the influence of distance on V1 responses is weaker when the visual drive is stronger.

A possibly similar effect was previously seen in a subset of V1 neurons selective to a rewarded position associated with a salient visual cue, when the cue was removed [4]. In our experiment, visual cues were stable, and yet the modulation by distance was seen, and in every position. Perhaps mice use different strategies in the two tasks: in our task, the reward zone was not indicated by a single visual cue but rather by the contingency of successive landmarks. Also, our animals were trained extensively (6–8 weeks), and perhaps this modulation is only revealed when animals take stereotyped paths in familiar environments [11].

It is not known how the distance traveled can modulate V1 neurons. Perhaps V1 estimates distance traveled by integrating visual speed and running speed [21], or perhaps it receives this estimate as a feedback signal from the navigation system, where self-motion signals abound. Feedback signals from the navigation system to visual cortex would likely involve intermediate areas such as retrosplenial cortex, which is connected to the hippocampal formation [22], sends dominant projections to V1 [23, 24, 25, 26], and encodes position and self-motion signals [27, 28, 29] that depend on the hippocampus [30].

Feedback signals from the navigational system to the visual system would explain our finding that activity of V1 is coupled to hippocampal theta oscillations. Such theta phase locking has been observed elsewhere in the cortex: in macaque visual area V4 during a working-memory task [31] and in rodent prefrontal and somatosensory cortices [16, 17, 18, 19]. Similar to the prefrontal cortex, we found that V1 interneurons were coupled to theta oscillations more often than pyramidal neurons. Moreover, our data also showed that the preferred theta phase of firing varied across cortical layers in V1.

We found that the phase of the theta cycle also shifted the positions encoded by V1. Theta phase precession has been observed beyond the hippocampus, e.g., in the entorhinal cortex [32, 33], ventral striatum [34], and prefrontal cortex [18, 19]. The theta phase coding that we found in V1 is smaller than in CA1, possibly reflecting a reduction of its effect through polysynaptic connections, or a convergence of signals oscillating at different theta phases. The phase of the spatial oscillation in V1, moreover, was often opposite to that observed in dorsal CA1. Perhaps V1 is more influenced by ventral CA1 or by CA3, where theta phase precession is out of phase in relation to dorsal CA1 [35, 36]. Alternatively, the spatial shift in V1 responses across theta phases might also be generated *de novo* within V1. We found that neurons in deep layers fire more often at a theta phase of ∼80° and superficial neurons at ∼210°. A delayed combination of such signals across layers might generate a spatial oscillation that is out of phase from CA1.

Why should the spatial representation in V1 be modulated by these non-visual signals? Perhaps V1 is part of a network that transforms sensory information from eye-centered to world-centered coordinates and estimates the animal’s spatial location [37]. Although activity in the visual cortex has a strong visual bias compared with that of other regions, it combines an estimate of position based on visual evidence and an estimate of position based on non-visual cues such as the distance traveled from a previous visual location [2, 5]. Similar to a Kalman filter [38], self-motion information in visual cortex might thus synergize with responses imposed by external sensory inputs to generate a more accurate estimate of position.

## STAR★Methods

### Key Resources Table

**Table:** 

REAGENT or RESOURCE	SOURCE	IDENTIFIER
**Deposited Data**		

Preprocessed data	This paper	https://doi.org/10.17632/jk4ggjysgp.1

**Experimental Models: Organisms/Strains**		

Mouse: C57BL/6J	https://www.jax.org/strain/000664	RRID: IMSR_JAX:000664

**Software and Algorithms**		

MATLAB	MathWorks	N/A
Klusta	[39]	https://github.com/kwikteam/klusta
Eye tracking	[40]	https://github.com/MouseLand/facemap
PsychToolBox	PsychToolBox	http://psychtoolbox.org
Virtual Reality Environment	[5]	https://github.com/amansaleem/SaleemLab-VR

### Resource Availability

#### Lead Contact

Further information and requests for resources should be directed to and will be fulfilled by the Lead Contact, Julien Fournier (julien.fournier@inserm.fr).

#### Materials Availability

This study did not generate new unique reagents.

#### Data and Code Availability

The pre-processed data used to generate the figures have been deposited to Mendeley Data: https://doi.org/10.17632/jk4ggjysgp.1

Raw data and code used to preprocess the data are available on request.

### Experimental Model and Subject Details

All experimental procedures were performed in accordance with the UK Animal Scientific Procedure Act 1986, under project and personal licenses issued by the UK Home Office.

Data were collected from ten C57BL/6J male mice (https://www.jax.org/strain/000664). Mice underwent surgery at 4-10 weeks of age. They were kept on 12-h light: 12-h dark cycle. Most animals were housed 2 per cage after surgery.

### Method Details

#### Surgical procedure

The surgical procedure is similar to that described previously [5]. In brief, mice were implanted on their left hemisphere with a 4-mm diameter chamber, under deep isoflurane anesthesia. Mice were left to recover for 3 days during which they received anti-inflammatory drug (Carprofen/Rymadil, administered orally). After recovery, mice were water-restricted and moved to light-shifted conditions (9 am light off, 9 pm light on). Mice were then trained once a day during their dark cycle, approximately at the same hour of the day, for several weeks. After they reached sufficient performance in the task, we performed two 1-mm craniotomies under deep isoflurane anesthesia: one over CA1 (1.0 mm lateral, 2.0 mm anterior from lambda), and the other over V1 (2.5 mm lateral, 0.5 mm anterior from lambda). Recordings were carried out on the subsequent 5-8 days with one recording session per day. Between recordings, the chamber was covered with silicon (KwikCast, World Precision Instrument).

#### Electrophysiological recordings

Recordings were performed acutely, with multi-site silicon probes connected to an amplifier (Blackrock Microsystems LLC) sampling at 30 kHz. Neurons in the dorsal CA1 region of the hippocampus were recorded using a silicon probe with 32 electrodes arranged in 8 tetrodes spread over four shanks (2 tetrodes per shank spaced by 150 μm vertically, 200 μm distance between shanks, NeuroNexus A4x2-tet). The pyramidal layer of CA1 was detected by the increase in power of theta oscillations (6–9 Hz) and the large number of detectable units. Neurons in primary visual cortex were recorded using a 32-electrode linear probe (20 μm between electrodes; NeuroNexus A1x32-Edge). In V1, the probe was inserted so that the most superficial electrode was ∼150 μm under the cortical surface. V1 and CA1 were recorded simultaneously in 35 sessions (7 mice, 4 to 6 sessions each).

The broadband signal was high-pass filtered at 500 Hz, and spike-sorted using Klustakwik and Klustaviewa [39]. We identified 2,748 neurons in CA1 (out of 54 recording sessions, 10 mice) and 1,433 neurons in V1 (out of 35 recording sessions, 7 mice). Hippocampal interneurons were identified based on the duration of their spike waveform (trough to next peak < 600 μs) and the shape of their spike time auto-correlogram (no prominent peak between 3-8 ms) [6]. All identified interneurons were excluded from further analysis. Single units in V1 were classified as putative interneurons if the duration of their spike waveform was < 600 μs from trough to next peak (21.7% of neurons; Figures S1D and S1E) [16, 41]; the remaining neurons were likely to be pyramidal.

Recording depth of V1 neurons from different sessions were registered by finding the depth at which the high frequency power (500 Hz–5 kHz) was highest (Figures S1F and S1G). The depth of this peak was at 596 ± 49 μm (MAD) below the cortical surface, consistent with the expected location of Layer 5 [42]. Most neurons that could be isolated were found in deep layers (Layer 4–6), consistent with previous studies using extracellular recordings [21, 29]. Neurons were sorted according to recording depths corresponding to putative cortical layers [42]: superficial neurons whose recording depths was between 250 μm and 500 μm (putative layer 2–4); intermediate neurons between 500 and 700 μm (putative layer 5) and deep neurons between 700 and 950 μm (putative layer 6). Almost all neurons in putative layer 6 were recorded above 900 μm; it is thus unlikely that they correspond to CA1 oriens or subiculum neurons which lie at depth > 1100 μm from the cortical surface.

We performed statistical tests across sessions rather than across mice, as the recordings were performed acutely (by re-inserting the electrode array) each session.

#### Local field potential and theta oscillations

Hippocampal local field potential (LFP) was extracted by filtering the broad-band signal between 0.1 Hz and 100 Hz. The LFP signal from the tetrode with the largest number of pyramidal neurons was filtered between 6 Hz and 9 Hz to obtain the theta-band oscillation. The instantaneous phase of the theta oscillation was measured by detecting the peaks in the oscillation and measuring the relative time from one peak to the next, which we then converted into phase. Peaks which occurred earlier than 60 ms after the preceding one were discarded. Theta phase values were centered so that the overall firing rate of the population of CA1 pyramidal neurons peaked at 180° theta phase.

#### Virtual environment and behavior

Mice navigated a virtual environment by walking on a cylindrical wheel made of polystyrene [5]. The movement of the wheel was measured with a rotary encoder (2,400 pulses per rotation, Kübler, Germany). Distance traveled on the wheel was used to translate the virtual environment presented on three LCD monitors (Hanns-G, 60 Hz refresh rate) placed in front of the animal at 34-cm viewing distance. To correct for luminance drop-off, Fresnel lenses were placed in front of the monitors. The eye position and pupil size were monitored with an infrared camera (DMK21BU04.H, Imaging Source) and a zoom lens (MVL7000, Navitar) at 25 Hz. Eye tracking was performed offline by fitting an ellipse to the pupil and measuring the center of mass and the area of this ellipse [40].

The virtual environment was a circular track (16 cm in width) made of two identical 200-cm semicircular corridors (Figure 1A). The main advantage of using a continuous circular track is that it avoids edge-effects at the start and the end of the animal’s trajectory. The track was covered with a 16.7-cm periodic grating. A wall ran along the right side of the track and was adorned with a periodic Gaussian-filtered white noise which repeated every 16.7 cm. The left side of the track had no wall. Three landmarks (L1, L2, L3) made of 24-cm long cylindrical tunnels (16 cm in diameter) were placed along the corridor and centered at 0 cm (L1), 83 cm (L2) and 117 cm (L3). The inside surface of these tunnels was covered with either a vertical grating (L1, L3) or a plaid (L1, L2). The outside surface of the tunnels was covered with a horizontal grating. Mice could navigate this environment indefinitely and there was no visual discontinuity going from one semicircular corridor to the next one, except for short interruption every 10 to 30 trials with a blank screen of several seconds (8.5 ± 2.5 s (s.d.)).

Mice were trained to lick selectively in a region centered around landmark L1 (±12 cm). Licks were detected using a custom-made infrared detector. When licks were detected in the correct region, the animal was rewarded with ∼2 μL water by opening of a pinch valve (NResearch, USA). To ensure that the animal did not learn the task by simply discriminating the visual texture associated with landmark L1, the visual texture displayed at landmark L1 alternated between a grating and a plaid every other trial.

Mice usually licked in bouts that we detected by identifying succession of licks that were spaced by less than 20 cm in the virtual corridor. We then labeled licks as being correct or incorrect depending on whether they were part of a bout of licks that overlapped with the reward region and resulted in reward delivery. The criterion for “correct” licks was independent of the gain condition.

Trials where the animal obtained the reward and made < 5 incorrect licks were considered as ‘correct’ trials. Trials where the animal made > 5 incorrect licks were labeled as ‘early’ if the previous trial was correct and ‘late’ if the animal missed the reward in the previous trial. The probability density of lick positions was computed from all licks that occurred before the reward delivery, considering only correct trials and smoothed with a Gaussian window (8 cm s.d.).

Mice typically learned this task in ∼6–8 weeks, i.e., they eventually licked exclusively when approaching the reward region and nowhere else in more than 80% of the trials. Once the animal had learned the task, we changed the gain of the virtual environment on a fraction of trials (36% ± 13 s.d.). On trials where the gain was lower (0.8), mice had to run 250 cm to reach the reward zone; on trials where the gain was higher (1.2), they had to run 166 cm to the reward zone. Gain changes were made in blocks of 5–10 trials. This manipulation was typically introduced 2–3 days before recording. Mice performed correctly on more than 70% of the trials when the gain was low or high.

We only considered time points when the run speed was > 5 cm.s^−1^, except when comparing lick positions.

### Quantification and Statistical Analysis

#### Spatial response profiles

Spike times were resampled at the screen refresh rate (60 Hz) and the position of the animal was binned into 2-cm bins. Trials were sorted according to the gain of the virtual environment and response profiles were computed separately on medium, low and high gain trials. The spatial response profile of each neuron was computed by measuring spike counts and occupancy as a function of position. The spike count map and the occupancy map were circularly smoothed with a Gaussian window (8-cm s.d.). Spatial response profiles were defined as the ratio of the smoothed spike count map over the smoothed occupancy map.

To test for the significance of response profiles, we circularly shifted in time each neuron spike train 500 times by a random period > 5 s. Neurons were considered to have a significant modulation of their firing rate by position if the maximal amplitude of their response profile was higher than the 99th percentile of the distribution of response amplitude computed from the shuffled controls. Neurons were then ordered according to their preferred position, defined as the position of maximal firing rate.

To estimate the shift in response profiles across gain conditions, we estimated the spatial offset by which the response profile measured at medium gain should be shifted to maximize the Pearson’s correlation with the response profile measured at low or high gain. To test for the significance of the spatial shift, we shuffled the gain labels associated to the spikes of each neuron 500 times. Neurons were considered to have a significant shift of their response profile on low or high gain trials when the absolute amplitude of this shift was larger than the 95th percentile of the distribution of shifts measured from the shuffled controls.

We also quantified the effect of the distance traveled by measuring how much variance the observed spatial shifts accounted for in the low or high gain response profiles (Figures S3E–S3I). To account for changes in mean firing rate, we fitted the low or high gain response ($R_{g}$) with the medium gain response profile ($R_{m}$) and measured the percentage of variance explained in the response profile (Equation 1) as follows:

$$\%Var=\frac{\sum_{x}{\left(R_{g}\left(x\right)-\alpha R_{m}\left(x\right)\right)}^{2}}{\sum_{x}{\left(R_{g}\left(x\right)-\langle Rg\;\left(x\right)\rangle_{x}\right)}^{2}}$$

where α corresponds to the fitted change in response amplitude, x corresponds to spatial positions, and <>x denotes the mean across positions.

We then measured the additional variance explained when we shifted the medium gain response profile with the spatial shift measured in the low or high gain conditions.

We also measured mean firing rates as a function of gain. To test if changes in gain significantly affected mean firing rates, we circularly shifted in time each neuron’s spike train 500 times. The mean firing rate was considered to be significantly different between medium and low or high gain trials, if the measured difference in firing rate was higher than the 99th percentile of the distribution of the same difference computed from the shuffled controls.

We also computed the response profile of each neuron as a function of position along the two successive semicircular corridors, which only differed by the presence of a grating or a plaid at landmark L1 (Figures 1A and S1A–S1C). We measured the difference in peak firing rate between the second and the first corridor at matching positions (i.e., 200 cm apart). To test if the difference in peak firing rate was significant, we shuffled 500 times the identity of the semicircular corridor (first versus second). A neuron was considered to respond differently in the first and second corridor if the difference in peak firing rate was larger than the 99^th^ percentile of the distribution of the same difference computed from the shuffled controls.

#### Theta modulation of firing rates

Firing rate as a function of the phase of theta oscillations was computed similar to spatial response profiles. Theta phases were binned into 20° bins. We measured the spike counts and occupancy as a function of the phase of the theta oscillation. The spike count map and the occupancy map were circularly smoothed with a Gaussian window (40° s.d.). The average firing rate across theta phases was defined as the ratio of the smoothed spike count map over the smoothed occupancy map. The preferred theta phase of firing was measured for each neuron from the position of the peak. The theta modulation index was computed as (Equation 2):

$$Theta\;modulation\;index=\frac{F_{max}-F_{min}}{F_{mean}}\;$$

where *F*_*max*_ and *F*_*min*_ correspond to the maximum and minimum firing rate across theta phases; *F*_*mean*_ is the mean firing rate across all theta phases.

We also quantified the effect of theta oscillations by measuring how much variance the theta modulation of firing rates accounted for in the 2D response profiles across theta phases and positions (Figures S4F–S4H). For each neuron, we fitted the *theta phase x position* response profile with the spatial response averaged across all phases and measured the additional variance explained when the gain of the spatial response was allowed to change across theta phases.

To measure the significance of theta modulation of firing rate, we circularly shifted in time each neuron’s spike train 500 times by a random period > 5 s. Firing rate was considered significantly modulated by theta phase if its theta modulation index was higher than the 95th percentile of the distribution of theta modulation index computed from the shuffled controls.

The fraction of V1 neurons modulated by theta phases was computed for three ranges of recording depths (putative layer 2–4: 250 to 500 μm; putative layer 5: 500 to 700 μm; putative layer 6: 700 to 950 μm). To account for possible sampling biases across animals, the significance of the dependence of the fraction of neurons across cortical layers was calculated by shuffling 500 times the neurons’ recording depths within animals rather than across the entire population. The fraction of theta-modulated neurons was considered to be significantly different between cortical layers when this difference was larger than the 95th percentile of the distribution of absolute differences measured from the shuffled controls.

To estimate the significance of the difference between distributions of preferred theta phases in different layers, we used a circular analog of the Kruskal-Wallis test (multi-sample test for equal circular medians). The test statistic was computed as defined in [43] and compared to the distribution obtained by shuffling 500 times the neurons’ recording depths within animals.

We also compared our results to those obtained with the pairwise phase consistency value (PPC) [44]. PPC values were estimated for each neuron as (PPC, Equation 3):

$$PPC=\frac{2}{N\left(N-1\right)}\sum_{i=1}^{N-1}\sum_{j=i+1}^{N}\left(\cos\left(\theta_{i}\right)\cos\left(\theta_{j}\right)+\;\sin\left(\theta_{i}\right)\sin\left(\theta_{j}\right)\;\right)$$

where N is the total number of spikes and θ_*i*_ corresponds to the theta phase of the *i*^th^ spike. To test for the significance of pairwise phase consistency values, we used a similar bootstrap approach as the one used for the theta modulation index: neurons were considered to have a significant preferred theta phase of firing when their PPC index was larger than the 95th percentile of the distribution of PPC values computed from the shuffled controls. As previously reported [44], the theta modulation index and the PPC values were closely related to each other: *PPC ∝* (Thetamodulationindex)^2^. The PPC method indeed gave similar results to the method based on the theta modulation index: 90.8% of CA1 neurons and 23.9% of V1 neurons had a significant PPC value; the relationship between theta modulation and cortical layers was also significant when using statistics based on PPC values.

#### Theta phase dependence of spatial responses

To assess how spatial selectivity changed as a function of theta phase, we measured average firing rates as a function of both the position of the animal and the phase of the theta oscillation. Positions were binned in 2-cm bins and theta phase into 20° bins. Spike count and occupancy were measured for each position and theta phase bin. The spike count map and the occupancy map were circularly smoothed using a 2d-Gaussian window (40° x 8-cm s.d.). The *theta phase* x *position* response profile was defined as the ratio between the spike count and occupancy maps. We then measured the spatial cross-correlogram between the response profile estimated at each theta phase with the mean response profile averaged across all phases (Figures S4K and S4L). The spatial drift of the response profile across a theta cycle was defined as the maximum of this cross-correlogram across theta phases. This maximum position curve was fitted with a sinusoid from which we measured the theta phase offset (i.e., the phase of zero-crossing) and the amplitude.

We also estimated the effect of theta oscillations on spatial selectivity by measuring how much variance the spatial shift observed across theta phases accounted for in the 2D response profiles across theta phases and positions (Figures S4F, S4I, and S4J). We first fitted the *theta phase x position* response profile with the mean spatial response, whose gain was adjusted for each theta phase to account for the theta-modulation of firing rate. We then quantified the additional variance explained when the mean spatial profile was spatially shifted across a theta cycle as measured from the spatial cross-correlogram.

To measure the significance of the spatial drift across theta phases, we shuffled the theta phases of the spikes of each neuron 500 times. Neurons were considered to have a significant spatial drift when the amplitude of the drift measured from the spatial cross-correlogram was higher than the 95th percentile of the distribution of spatial drifts measured from the shuffled controls. To make sure that the measured drift was not an artifact of smoothing across phases, significance levels were calculated without smoothing the *theta phase* x *position* response profiles along the phase dimension.

The fraction of V1 neurons whose response profile was significantly shifted across theta phases was computed for three ranges of recording depths (putative layer 2–4: 250 to 500 μm; putative layer 5: 500 to 700 μm; putative layer 6: 700 to 950 μm). To test for the significance of the difference in the fraction of theta-shifted neurons or the median of the theta phase offset across cortical layers, we used a bootstrap method similar to the one used for the modulation of firing rates across theta phases.

For comparison, we also estimated the relationship between the theta phase and position of spikes by estimating the linear-circular correlation [45]. With this metric, neurons were considered to show a significant association between theta phase and position when the absolute value of their linear-circular correlation coefficient was larger than the 95th percentile of the distribution of correlation coefficients measured from the shuffled controls. Using this method, we found similar fractions of response profiles that were spatially shifted across phases of a theta cycle (CA1: 26.6%; V1: 9.0%). However, the linear-circular correlation is not only sensitive to the shift in the spatial profile across a theta cycle; it also depends on the static modulation of the firing rate by theta phases, which interferes with its ability to reflect the amplitude of the spatial shift across theta phases. For instance, the linear-circular correlation can sometimes be positive in place cells whose response profile is shifted in the direction expected from classical phase precession (see Figure S4K). Moreover, the linear-circular correlation method requires to identify the peak and the extent of the place field. The spatial correlogram method that we implemented thus appeared more sensitive than the classical linear-circular correlation to quantify the spatial shift across theta phases.

#### Bayesian decoding

To decode positions from populations of simultaneously recorded neurons, we used an independent Bayes decoder, assuming spike times followed a Poisson distribution. Decoding was performed for recording sessions where > 10 neurons showed a significant modulation of their firing by position (CA1, n = 40 sessions; V1, n = 32 sessions; n = 27 sessions with simultaneous recording). For every time bin, we estimated the probability P(x|R)of the animal being at a given location from the spike count of CA1 or V1 neurons, using the following formula (Equation 4) [46]:

$$P\left(x|R\right)=\frac{1}{Z}P\left(x\right)\left(\prod_{i=1}^{M}f_{i}{\left(x\right)}^{r_{i}}\right)exp\left(-t\sum_{i=1}^{M}f_{i}\left(x\right)\right)$$

Where *x* is the position of the animal, *r*_*i*_ is the spike count of cell *i*, *f*_*i*_*(x)* is the spatial response profile of cell *i* estimated as described above, *M* is the total number of neurons, *t* is the size of the spike counting window and *Z* is a normalizing constant that ensures that the resulting distribution sums to 1. Similar results were obtained when using a look-up table to estimate P(x|R) instead of assuming a Poisson distribution of spike counts.

The response profiles *f*_*i*_*(x)* were computed from medium gain trials as described above. The spike count window was fixed to 250 ms (except when we assessed the influence of theta phases on decoded positions where we used 50 ms; Figures 4H, 4K, and S4T). To avoid over-fitting, the posterior probability decoded from medium gain trials was computed using a 20-fold cross-validation procedure: response profiles were calculated from 95% of the data and the posterior probability was estimated from the spike counts recorded in the remaining 5%; this procedure was repeated iteratively on different subsets until all trials were decoded.

The decoded position at each time point was estimated as the position of maximum of the posterior probability distribution P(x|R). The probability density of decoded positions as a function of the actual position of the animal (Figures 1E, 3A, and 3F) were calculated as the distribution of decoded positions in each position of the animal across trials, which was normalized to sum to 1. We smoothed the joint-distribution with a 2d-Gaussian window (2 × 2-cm s.d.). The decoding error in each time point was computed as the circular distance between the decoded position and the actual position of the animal. We also computed the distribution of decoding errors as a function of the position of the animal. The probability density of decoding errors (Figures 3B and 3G) was computed by averaging the distribution of decoding errors in each position of the animal which was normalized to sum to 1. The mean decoding error (Figures 3C and 3H) was defined as the weighted circular average of this probability distribution. For each session, the mean decoding errors measured at low and high gain were corrected by the mean decoding error obtained at medium gain. We also measured the most-likely decoding error (*decoding bias*, Figures 3D and 3I) as the maximum of the distribution of decoding errors in each position. For low and high gain, the decoding bias as a function of positions was corrected by the decoding bias measured on medium gain trials. The standard error of the distribution of decoding errors and of the decoding bias across sessions was estimated using a 20-fold Jackknife resampling procedure.

To estimate the mean decoding error as a function of theta phases, we decoded positions using a 50-ms spike count window. Theta phases were binned into 20° bins and the mean decoding error was computed for each phase bin as described above. The resulting curve was smoothed across theta phase with a Gaussian window (40° s.d.). The amplitude and phase offset of this shift in decoding error across theta phase was measured from the best fitting sinusoid. To test for the significance of this shift, we randomized theta phases 500 times. Decoding errors were considered as significantly influenced by theta when the amplitude of the shift across theta phases was larger than the 95th percentile of the shifts measured from the shuffle controls.

#### Correlations between V1 and CA1 errors

Correlations between V1 and CA1 decoding errors (Figures S2B–S2D) were estimated from correct trials as previously described [5]. For each position and speed bin, we calculated the joint distribution of V1 and CA1 decoding errors, smoothed with a 2d-Gaussian window (4 × 4-cm s.d.). To account for the contribution of position and speed to the covariance between V1 and CA1 errors, we computed the same distribution after shuffling time points when the animal was in the same position (within 2 cm) and run at the same speed (binned in 5 quantiles of the speed distribution). The result of the subtraction between the measured and shuffled distributions gave an estimate of the covariance between V1 and CA1 errors that could not be accounted for by positions or speed. A similar analysis was performed on the distribution of mismatches between V1 and CA1 decoded positions, defined as the circular distance between decoded positions (Figures S2E and S2F).

To test whether the correlation between V1 and CA1 decoding errors could result from a common overfit of spurious behavioral events that would influence firing rates in both areas, we split the trials of each session in three equal parts. We then used the first part to train the V1 decoder; the second part to train the CA1 decoder and the third part to measure the covariance between V1 and CA1 errors (Figure S2C).

The theta modulation of decoding errors was estimated as the absolute amplitude of the sinusoid that best fitted decoding errors over each theta cycle. We then measured the Pearson’s correlation between the amplitudes of this modulation measured from V1 and CA1 across theta cycles (Figure S4T) and compared it to the correlation obtained after shuffling time points as described above.

#### Generalized linear model

To test for the possible contribution of behavioral fluctuations to the measured effect of the distance traveled on spatial profiles (Figures S3J–S3O), we used a multilinear ridge regression model to estimate the contribution of speed, acceleration, pupil size, pupil position, licks and reward to the spike count of each neuron (250-ms spike count window). The model parameters were estimated from responses at medium gain trials. Since the animal’s behavior and its position along the track were strongly correlated, we used a two-step fitting procedure which ensured orthogonality between the positional and behavioral components of the model.

In the first step, we estimated the contribution of positions (within 2 cm) to the response on medium gain trials (Equation 5):

$$y\left(t\right)=\sum_{i=1}^{100}\beta_{i}X_{i}\left(t\right)$$

Where y(t) is the mean centered spike count at time t and X_i_(t) are 1 if the animal was at position i at time t and 0 otherwise. The model prediction ${\hat{y}}_{pos}\left(t\right)\;$was computed for all trials (using a 20-fold cross-validation procedure for medium gain trials).

In the second step, behavioral variables were smoothed with a 250-ms time window and binned in 10 quantiles. We then fitted the contribution of these variables to the residual part of the response which could not be explained by position (Equation 6):

$$y\left(t\right)-{\hat{y}}_{pos}\left(t\right)=\sum_{\tau \;\in \;\left[-1:0.25:1\right]}\left(\sum_{i=1}^{10}\beta_{s_{i}}\left(\tau \right)S_{i}\left(t-\tau \right)+\sum_{i=1}^{10}\beta_{A_{i}}\left(\tau \right)A_{i}\left(t-\tau \right)+\sum_{i=1}^{10}\beta_{P_{i}}\left(\tau \right)P_{i}\left(t-\tau \right)+\sum_{i=1}^{10}\beta_{ex_{i}}\left(\tau \right)Ex_{i}\left(t-\tau \right)+\sum_{i=1}^{10}\beta_{ey_{i}}\left(\tau \right)Ey_{i}\left(t-\tau \right)+\sum_{i=1}^{10}\beta_{L_{i}}\left(\tau \right)L_{i}\left(t-\tau \right)+\beta_{R}R\left(t-\tau \right)\right)$$

where τ is a time shift that varied between −1 and 1 s in steps of 250 ms and *S*_*i*_(*t*), *A*_*i*_(*t*), *P*_*i*_(*t*), *Ex*_*i*_(*t*), *Ey*_*i*_(*t*), *L*_*i*_(*t*) and *R*(*t*) are state variables corresponding to run speed, acceleration, pupil size, pupil azimuth, pupil elevation, number of licks and reward respectively and are 1 if the corresponding behavioral variable was in the ith bin at time t and 0 otherwise.

The response predicted in this second step ${\hat{y}}_{beh}\left(t\right)$was computed for all trials (using a 20-fold resampling procedure for medium gain trials). We then obtained the full model prediction $\hat{y}\left(t\right)$ by summing the positional and behavioral predictions:

$$\hat{y}\left(t\right)={\hat{y}}_{pos}\left(t\right)+{\hat{y}}_{beh}\left(t\right)$$

For each neuron, response profiles at low medium and high gain were computed from this prediction. Spatial shifts across gain conditions were estimated as described above, i.e., by selecting the spatial offset that maximizes the Pearson’s correlation with the medium gain response profile. We then compared the spatial shifts measured from the predicted response profiles to those measured from the original spike count response (Figure S3K). We obtained similar results when the contribution of behavioral variables was estimated from all gain conditions instead of just medium gain trials.

To estimate the optimal delay in neural response that could explain the spatial shift across gain conditions (Figures S3L–S3O), we fit the same model with positions shifted in time in the first step of the fitting procedure, while the second step remained identical (Equation 7):

$$y\left(t\right)=\sum_{i=1}^{100}\beta_{i}X_{i}\left(t-delay\right)$$

We explored delays from 0 to 1,000 ms in steps of 100 ms. The optimal delay was estimated as the one that resulted in the largest Pearson’s correlation between response profiles predicted at low or high gain and at medium gain (Figures S3N and S3O). The spatial shifts measured from the corresponding models were then compared to those measured from the original spike count response (Figure S3L).

## Supplemental Information

Document S1. Figures S1–S6

Document S2. Article plus Supplemental Information
