# Supplementary material for: Mouse Visual Cortex Is Modulated by Distance Traveled and by Theta Oscillations
Source: Curr Biol. 2020 Oct 5;30(19):3811–3817.e6. doi: 10.1016/j.cub.2020.07.006 (PMC7544510; doi:10.1016/j.cub.2020.07.006)
Supplement: Document S1. Figures S1–S6 [file mmc1.pdf]

Current Biology, Volume *30*

Supplemental Information

Mouse Visual Cortex Is Modulated

by Distance Traveled and by Theta Oscillations

Julien Fournier, Aman B. Saleem, E. Mika Diamanti, Miles J. Wells, Kenneth D. Harris, and Matteo Carandini

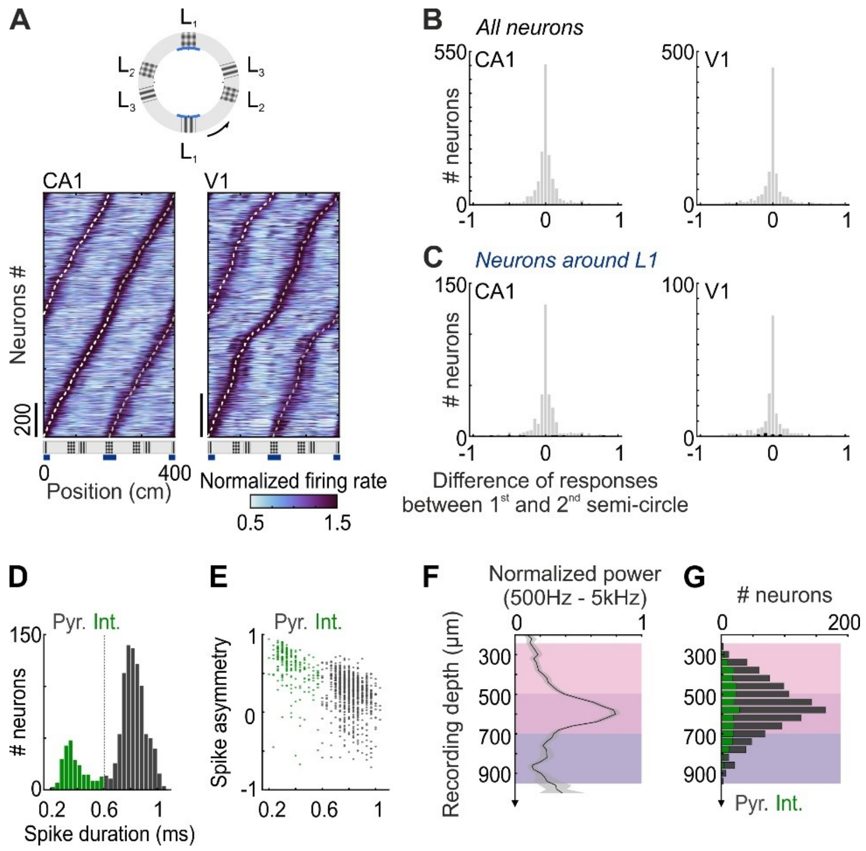

Figure S1. Environment geometry, V1 cell types and recording depth. Related to Figure 1.
A. *Top*: the virtual corridor was defined by three landmarks (L1, L2 and L3) and repeated in a full circle with texture at landmark L1 alternating between a grating and a plaid every other trial. *Bottom*: Response profiles of CA1 (*left*) and V1 (*right*) neurons estimated as a function of position along the two successive semicircular corridors, which differed by the texture associated with landmark L1 (grating vs. plaid). Neurons are ordered according to the position of their peak response. Matching positions in the two semicircular corridors (200 cm away) are indicated by the dashed lines. Although the texture at the reward zone (L1) alternated between a grating and a plaid, responses were very similar between the two corridors.

B. Distribution of the difference between peak responses in the first (starting with a grating, *white line* in B) and second (starting with a plaid, *gray line* in B) corridor, for CA1 (*left*) and V1 (*right*) neurons. The difference is expressed relative to the mean firing rate across all positions. *Black bars*: neurons with significant difference in peak firing rate between the first and second corridor. Only 1.0% of V1 neurons (0.8% in CA1) showed a significant difference in peak firing rate between the first and second corridor (V1: n = 1127 neurons; CA1: n = 1431 neurons; $p < 0.01$).

C. Same as **C** after selecting only neurons which had a maximal firing rate within 40 cm around landmarks L1. Even neurons which fired maximally around landmarks L1 were minimally affected by the change of texture from a grating to a plaid between the two corridors (CA1, 2.3%, n = 350 neurons; V1, 2.4%, n = 212 neurons; $p < 0.01$).

D. Distribution of the duration of spike waveforms (trough to next peak; high-pass cutoff, 500 Hz) across V1 neurons. Putative neocortical interneurons and pyramidal cells were isolated from the duration of their

extracellular spike waveform [S1,S2] (*Green*, putative interneurons; *Gray*: putative pyramidal neurons; threshold: 0.60 ms).

E. Other metrics such as the spike asymmetry [S2] did not help to better separate putative interneurons and pyramidal cells in our dataset.

F. Normalized V1 LFP power between 500 Hz and 5 kHz as a function of recording depth, averaged across V1 sessions (n = 35). *Gray band* shows ± s.e.m. Recording depths across sessions were registered according to the peak power at high frequency, which most likely corresponds to layer 5 [S3].

G. Distribution of recording depth of all V1 neurons (*Green*, putative interneurons; *Gray*: putative pyramidal neurons).

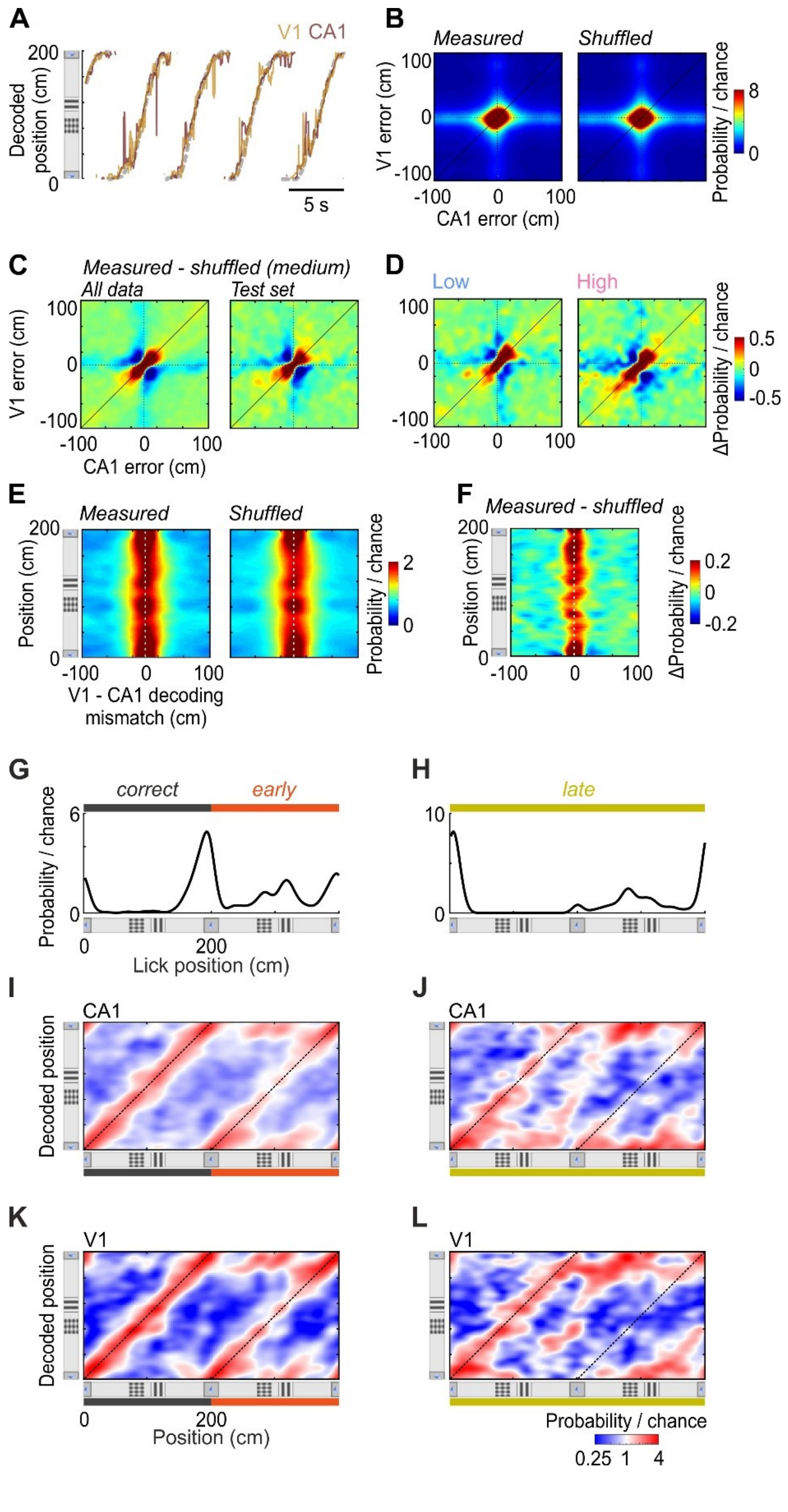

A
V1 CA1
200
Decoded position (cm)
0
5 s
B
Measured
Shuffled
100
0
-100
V1 error (cm)
-100 0 100
CA1 error (cm)
Probability / chance
8
0
C
Measured - shuffled (medium)
All data
Test set
D
Low
High
0.5
0
-0.5
ΔProbability / chance
E
Measured
Shuffled
Position (cm)
V1 - CA1 decoding mismatch (cm)
2
F
Measured - shuffled
0.2
-0.2
G
correct
early
6
Probability / chance
Lick position (cm)
H
late
10
I
CA1
Decoded position
J
K
V1
L
Position (cm)
Probability / chance
0.25 1 4

Figure S2. Correlations between V1 and CA1 decoding errors. Related to Figure 1.

A. Examples of positions decoded from V1 (*yellow*) and CA1 (*brown*) populations recorded simultaneously for one session example. *Dashed line*: actual position of the animal.

B. Joint distribution of V1 and CA1 decoding errors, averaged across spatial positions and sessions (n = 27). *Left*, joint distribution obtained from the actual data. *Right*, joint distribution obtained after shuffling time points within position and speed bins.

C. Difference between joint distributions of V1 and CA1 errors obtained from the actual data and from the shuffled control (measured - shuffled). *Left*, density map obtained from medium gain trials, with V1 and CA1 decoders being trained on the same dataset. *Right*, the medium gain trials were split into three parts: V1 and CA1 decoders were trained on two different thirds and the density map was obtained from the last third. This control shows that the correlation between V1 and CA1 errors cannot be explained by occasional behavioral events, unrelated to positions, which could potentially be overfit by V1 and CA1 decoders trained on the same dataset.

D. Same as in C. testing on low (*left*) or high (*right*) gain trials, with the decoders trained on medium gain.

E. Distribution of mismatches between V1 and CA1 decoding errors in all spatial positions, averaged across sessions (n = 27). *Left*, distribution obtained from the actual data. *Right*, distribution obtained after shuffling time points within position and speed bins.

F. The difference between V1 - CA1 mismatch distributions obtained from the actual data and from the shuffled control (measured - shuffled) does not show a trend across position.

G. Average distribution of lick positions on consecutive trials where the animal made mistakes by licking too early after completing a correct trial (‘early’ trials). Only medium gain trials were included in this analysis.

H. Same as in G. for consecutive trials, where the animal licked in the wrong location after missing the reward zone in the previous trial (‘late’ trials).

I. Distribution of positions decoded from CA1 as a function of the animal’s position on consecutive trials where the animal made mistakes by licking too early after completing a correct trial (‘early’ trials).

J. Same as in **I**. for consecutive trials, where the animal licked in the wrong location after missing the reward zone in the previous trial (‘late’ trials).

K-**L**. Same as in **I**-J, based on decoding from populations of V1 neurons.

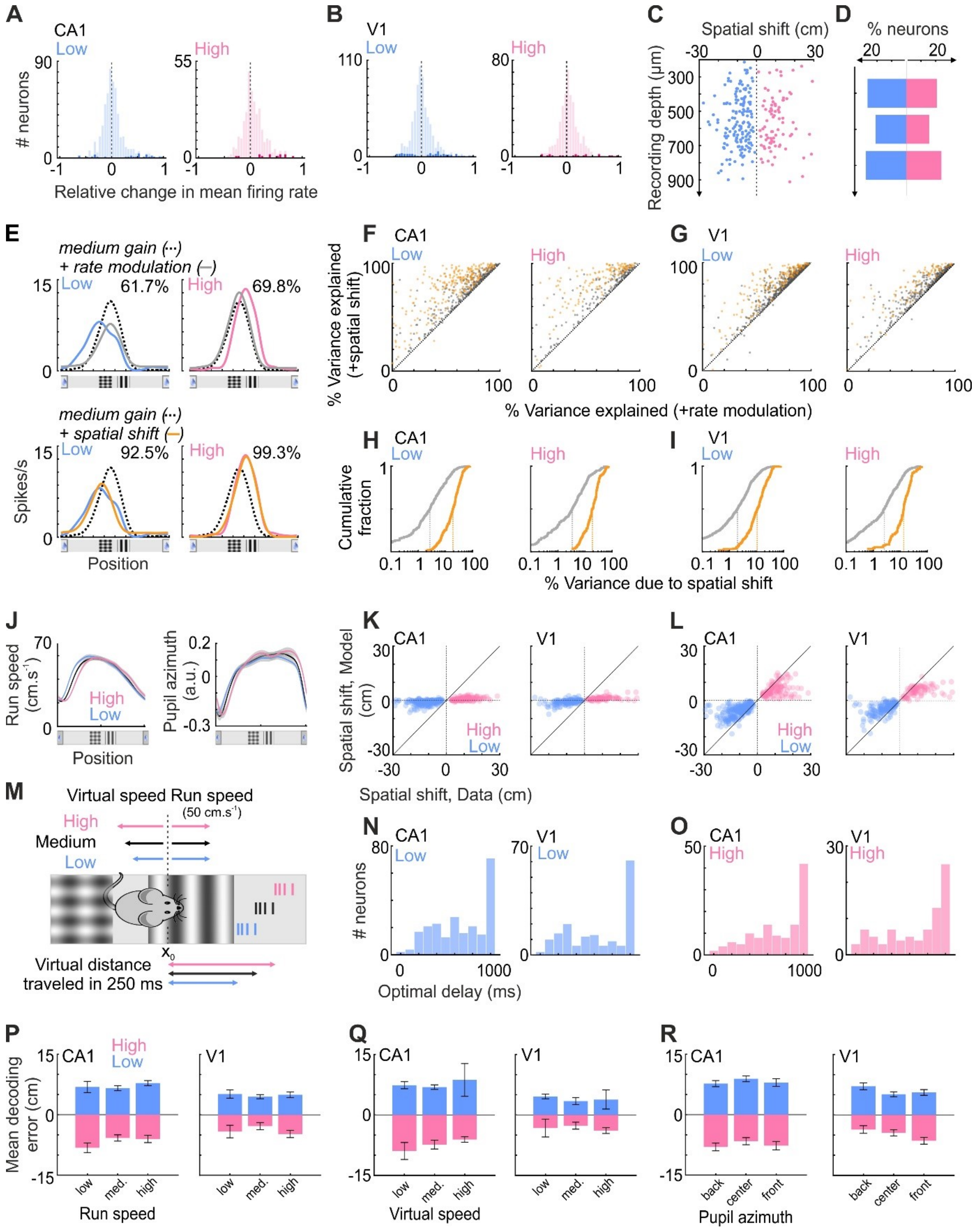
A
CA1
Low
High
neurons
Relative change in mean firing rate
B
V1
Low
High
C
Spatial shift (cm)
Recording depth (µm)
D
% neurons
E
medium gain (··)
+ rate modulation (—)
Low 61.7%
High 69.8%
medium gain (··)
+ spatial shift (—)
Low 92.5%
High 99.3%
Spikes/s
Position
F
CA1
G
V1
% Variance explained (+spatial shift)
% Variance explained (+rate modulation)
H
CA1
I
V1
Cumulative fraction
% Variance due to spatial shift
J
Run speed (cm.s-1)
Pupil azimuth (a.u.)
Position
K
CA1
V1
Spatial shift, Model (cm)
Spatial shift, Data (cm)
L
CA1
V1
M
Virtual speed Run speed
(50 cm.s-1)
High
Medium
Low
x0
Virtual distance
traveled in 250 ms
N
CA1
V1
neurons
Optimal delay (ms)
O
CA1
V1
P
CA1
V1
Mean decoding error (cm)
low
med.
high
Run speed
Q
CA1
V1
Virtual speed
R
CA1
V1
back
center
front
Pupil azimuth

Figure S3. Modulation of V1 and CA1 activity by the distance traveled. Related to Figure 2.

A. Distribution of the difference in mean firing rate between medium gain trials and low (*left*) or high (*right*) gain trials, for CA1 neurons which had a significant change in firing rate across positions (low gain, 559 neurons; high gain, 329). The difference was normalized by the mean firing rate on medium gain trials. Darker colors represent neurons which had a significant change in mean firing rate on low or high gain trials compared to medium gain (low gain, 6.1%; high gain, 5.8%; $p < 0.01$).

B. Same as **A** for V1 neurons with a significant change in firing rate across positions (low gain, 667 neurons; high gain, 446). Proportion of V1 neurons with significant mean rate change: low gain, 10.0%; high gain, 4.9%; $p < 0.01$).

C. Spatial shift in response profiles of V1 neurons detected at different depths. Only neurons with a significant spatial shift are represented ($p < 0.05$).

D. Percentage of neurons that showed a significant shift in response profiles at low or high gain at three ranges of recording depth (250-500μm, 500-700μm, 700-950μm).

E. *Top*: to quantify how much the spatial shifts observed at low and high gain contributed to the response profiles, we first measured how much the response at medium gain (*dashed*) could explain the response at low (*blue, left*) or high gain (*pink, right*) when adjusting for changes in mean firing rate (*gray*). In this example, the spatial modulation observed at medium gain could explain 61.7% of the variance of the spatial profile at low gain and 69.8% at high gain. *Bottom*: we then measured how much the response at medium gain (*dashed*) could explain the response at low (*blue, left*) or high gain (*pink, right*) when we additionally shifted in space the medium gain response. In the example shown, the spatial shift measured between medium and low gain responses could explain 92.5% of the variance of the spatial profile at low gain and 99.3% at high gain. For this neuron example, we thus concluded that the spatial shift due to the change in distance traveled accounted for an additional 30.8% of the variance of the response profile at low gain (92.5 - 61.7) and 29.5% at high gain (99.3 - 69.8).

F. Percentages of variance in CA1 spatial response profiles explained with spatial shift (y-axis, +spatial shift) or without (x-axis, +rate modulation), for low (*left*) and high gain (*right*). Responses at low and high gain were fitted as described in **E**. Each dot corresponds to one neuron. *Yellow*: neurons whose response profiles were significantly shifted at low or high gain ($p < 0.05$). *Gray*: neurons whose response profiles were not significantly shifted at low or high gain ($p > 0.05$).

G. Same as **F** for V1 neurons.

H. Cumulative distribution of the percentage of variance in low (*left*) and high (*right*) gain response profiles that was additionally explained by spatially shifting the medium gain response profiles (see **F**). *Yellow curve*: neurons whose response profiles were significantly shifted at low or high gain ($p < 0.05$). *Gray curve*: neurons whose response profiles were not significantly shifted at low or high gain ($p > 0.05$). Dashed lines indicate the median across neurons.

I. Same as **H** for V1 neurons.

J. Mean run speed (*left*) and pupil azimuth (*right*) as a function of positions in the corridor. Behavioral variables were similar across gain conditions, except at the start and the end of the corridor, reflecting the change in the licking behavior of the animal.

K. To test whether spatial shifts in response profiles could be explained by fluctuations in the behavior of the animal across gain conditions, we constructed a multilinear regression model whereby firing rate depends on the animal's position but also on run speed, eye position (azimuth and elevation), pupil size, licks and reward. This model was fitted for each neuron on medium gain trials. We then computed the response profile on low, medium and high gain trials from the predicted activity and measured the spatial shift between the predicted response profiles in the same way as for the actual data. Spatial shifts predicted by this model could not explain those measured from the actual data.

L. To test whether spatial shifts in response profiles could be explained by a temporal delay in neural responses (see **M**), we fitted the same multilinear regression model as described in **K** while shifting forward in time the position of the animal by different delays. We then estimated the 'optimal delay' as the one that maximizes the Pearson's correlation between response profiles predicted at medium and low or high gain. This model could still not explain the full range of spatial shifts measured from the data.

M. Example of spatial shift expected at low and high gain from a neuron responding with a 250-ms delay. Since mice ran at similar speeds across gain conditions, the virtual environment moved faster or slower depending on the gain. The spatial shifts in neural responses could thus potentially reflect a temporal delay in the response of neurons, which would translate into a displacement of their response in space at low or high gain. In this example, a neuron responding with a 250-ms latency when the mouse crosses position x_0 will appear to fire 2.5 cm earlier at low gain and later at high gain if the mouse runs at 50 $cm.s^{-1}$.

N. Distribution of the optimal delays that were estimated on low gain trials using the multilinear regression model described in **L**. The optimal delays that best fitted the observed spatial shifts in V1 responses on low gain trials are not compatible with the latency of visual responses commonly observed in V1 (median: 650 ms).

O. Same as **N**. for high gain trials. The optimal delays that best fitted the observed spatial shifts in V1 responses on high gain trials are not compatible with the latency of visual responses commonly observed in V1 (median: 750 ms).

P. Mean decoding errors measured in CA1 (*left*) and V1 (*right*) for three ranges of running speed. Speed ranges were defined as the deviation of the running speed from the median of the running speeds at the animal's current position on medium gain trials.

Q. Same as **P**. for virtual speed.

R. Same as **P**. for pupil azimuth.

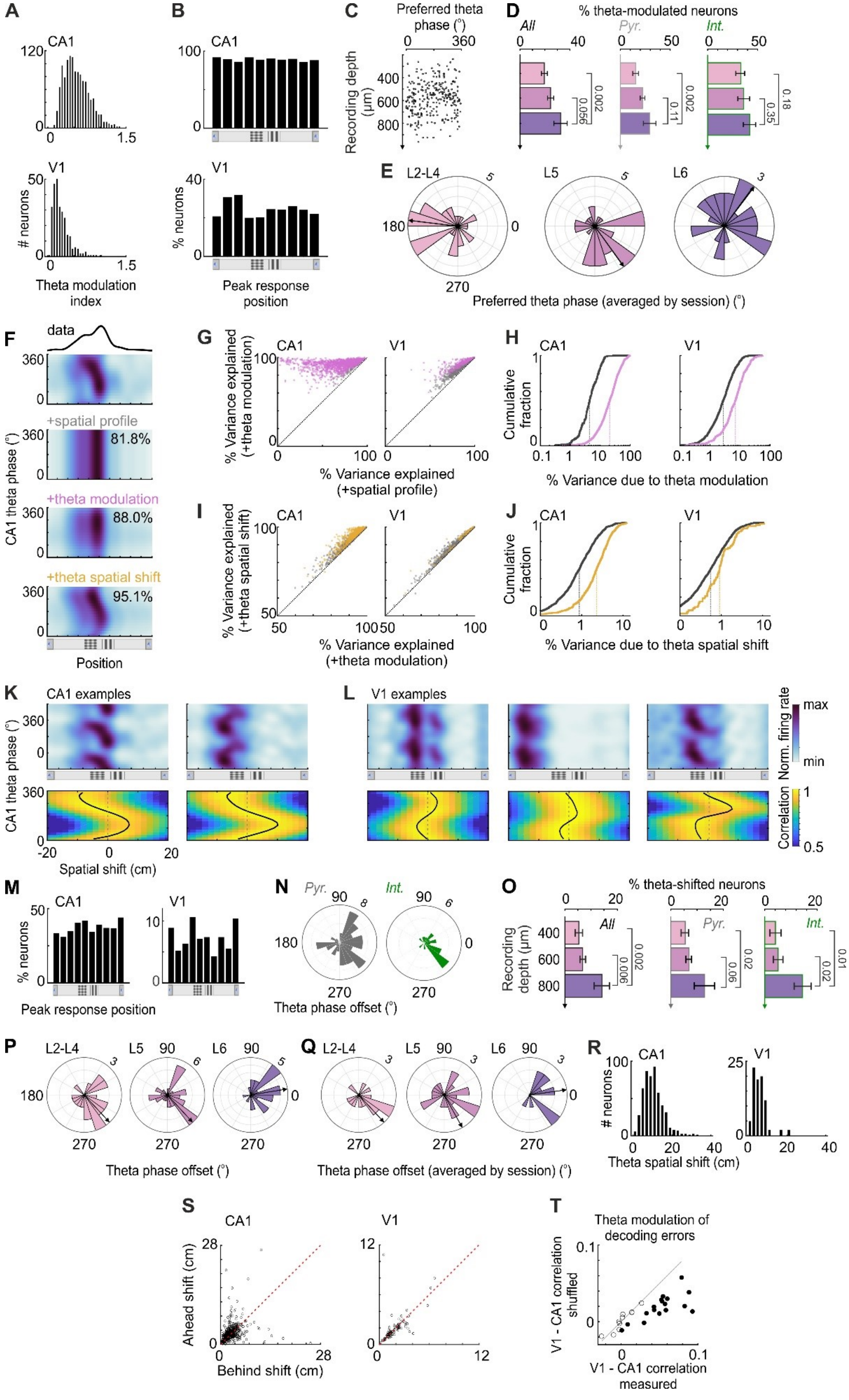

A
CA1
Theta modulation index
V1
neurons
B
% neurons
Peak response position
C
Preferred theta phase (°)
Recording depth (μm)
D
% theta-modulated neurons
All
Pyr.
Int.
E
L2-L4
L5
L6
Preferred theta phase (averaged by session) (°)
F
data
+spatial profile
81.8%
+theta modulation
88.0%
+theta spatial shift
95.1%
CA1 theta phase (°)
Position
G
% Variance explained (+theta modulation)
% Variance explained (+spatial profile)
H
Cumulative fraction
% Variance due to theta modulation
I
% Variance explained (+theta spatial shift)
% Variance explained (+theta modulation)
J
% Variance due to theta spatial shift
K
CA1 examples
Spatial shift (cm)
L
V1 examples
Norm. firing rate
max
min
Correlation
M
Peak response position
N
Theta phase offset (°)
O
% theta-shifted neurons
Recording depth (μm)
P
Theta phase offset (°)
Q
Theta phase offset (averaged by session) (°)
R
Theta spatial shift (cm)
S
Ahead shift (cm)
Behind shift (cm)
T
Theta modulation of decoding errors
V1 - CA1 correlation shuffled
V1 - CA1 correlation measured

Figure S4. Theta modulation of CA1 and V1 firing rates and spatial responses. Related to Figure 4.

A. Distribution of the amplitude of the firing rate modulation of CA1 (*top*) and V1 (*bottom*) neurons by theta phases (*Theta modulation index*). Only neurons with a significant modulation by theta are included.

B. Percentages of CA1 (*top*) or V1 (*bottom*) neurons with a significant modulation of their firing rate by theta ($p < 0.05$), as a function of the position of maximal firing along the corridor.

C. Theta phase of maximal firing as a function of recording depth for V1 neurons.

D. Percentages of neurons with a significant firing rate modulation by theta at three ranges of recording depth (250-500µm, 500-700µm, 700-950µm). *Left*: All neurons; *Middle*: putative pyramidal neurons; *Right*: putative interneurons. Error bars indicate standard errors across sessions, estimated by a leave-one-out Jackknife resampling method.

E. Distribution of the mean theta phase of maximal firing across recording sessions. The mean theta phase was obtained by averaging across neurons recorded during the same session, sorted by recording depth (L2-4: 250-500µm; L5: 500-700µm; L6: 700-950µm). *Arrow*: median of the preferred theta phase across sessions.

F. To quantify how much the modulation by the theta oscillation contributed to the response profile of V1 and CA1 neurons, we first measured how much of the variance in the 2D response profile (theta phase x position) could be explained by the mean spatial profile, without modulation of firing rate or position by theta phase (+*spatial profile*). In the example shown, the mean spatial profile could account for 81.8% of the variance of the response profile across theta phase and position. To quantify the contribution of the theta modulation of firing rates, we then measured how much variance in the 2D response profile could be explained when we additionally allowed the mean spatial profile to change in amplitude as a function of theta phases (+*theta modulation*). In the example shown, adding the theta modulation of firing rate accounted for an additional 6.2% of variance (88.0 - 81.8). Finally, we measured how much the spatial shift across theta phases additionally accounted for in the 2D response profile (+*theta spatial shift*). In the example shown, the theta-dependent spatial shift could explain 7.1% (95.1 - 88.0) more variance in the 2D response profile (*theta phase x position*) compared to when no theta spatial shift was allowed (+*theta modulation*).

G. Percentages of variance in the 2D response profiles (*theta phase x position*) that could be explained with the theta modulation of firing rates (*y-axis*, +*theta modulation*) or without (*x-axis*, +*spatial profile*), for CA1 (*left*) and V1 (*right*) neurons. The 2D response profiles (*theta phase x position*) were fitted as described in **F**. Each dot corresponds to one neuron. *Purple*: neurons whose firing rate was significantly modulated across a theta cycle ($p < 0.05$). *Gray*: neurons whose firing rate was not significantly modulated across a theta cycle ($p > 0.05$).

H. Cumulative distribution of the percentage of variance in the 2D response profiles (*theta phase x position*) that was additionally explained by adding theta modulation to the mean spatial profiles (see **F**). *Purple curve*: neurons whose firing rate was significantly modulated across a theta cycle ($p < 0.05$). *Gray curve*: neurons whose firing rate was not significantly modulated across a theta cycle ($p > 0.05$). *Dashed line*: median across neurons.

I. Percentages of variance in the 2D response profiles (*theta phase x position*) that could be explained with the theta-dependent spatial shift (*y-axis*, +*theta spatial shift*) or without (*x-axis*, +*theta modulation*), for CA1

(*left*) and V1 (*right*) neurons. The 2D response profiles (*theta phase x position*) were fitted as described in **F**. Each dot corresponds to one neuron. *Yellow*: neurons whose spatial profile was significantly shifted in space across a theta cycle ($p < 0.05$). *Gray*: neurons whose spatial profile was not significantly shifted in space across a theta cycle ($p > 0.05$).

J. Cumulative distribution of the percentage of variance in the 2D response profiles (*theta phase x position*) that was additionally explained by adding the spatial shift across theta phases to the mean spatial profiles (see **F**). *Yellow curve*: neurons whose spatial profile was significantly shifted in space across a theta cycle ($p < 0.05$). *Gray curve*: neurons whose spatial profile was not significantly shifted in space across a theta cycle ($p > 0.05$). *Dashed line*: median across neurons.

K. Examples of CA1 neurons with a significant spatial shift of their response profiles across theta phases ($p < 0.05$). For each neuron, we plotted the mean firing rate as a function of theta phases and positions (*top*) and the spatial cross-correlogram between the response profile at each theta phase and the mean spatial profile (*bottom*). Although both neurons exhibited a spatial shift typical of phase precession (ahead on late theta phases and behind on early theta phases), their linear-circular correlation coefficients were of opposite signs (*left*: -0.32; *right*: 0.14)

L. Same as in **K** for three examples of V1 neurons.

M. Percentages of CA1 (*left*) or V1 (*right*) neurons with a significant spatial shift of their response profile across theta phases ($p < 0.05$), as a function of the position of maximal firing along the corridor.

N. Distribution of the theta phase offset (phase of zero-crossing when the spatial shift went from positive to negative), for putative pyramidal cells (*left*, $p = 3.0\ 10^{-4}$, Rayleigh test) and interneurons (*right*, $p = 1.1\ 10^{-2}$; Rayleigh test) in V1. Only neurons with a significant spatial shift across theta phases are included (putative pyramidal cells, 7.4% of 882; putative interneurons, 8.1% of 245).

O. Percentage of V1 neurons (*left*: all neurons; *middle*: putative pyramidal cells; *right*: putative interneurons) that showed a significant modulation of their response profiles by theta phase at three ranges of recording depth (250-500µm, 500-700µm, 700-950µm). Error bars indicate standard errors across sessions, estimated by a leave-one-out Jackknife resampling method.

P. Distribution of the theta phase offset across neurons, sorted by recording depth (L2-4: 250-500µm; L5: 500-700µm; L6: 700-950µm). Only neurons with a significant spatial shift across theta phases are included. *Arrow*: median of the theta phase offset across neurons. The phase offset was significantly different between deep and intermediate layers but not superficial layers (L6 vs. L5: $p = 0.002$; L6 vs. L2-4: $p = 0.48$).

Q. Distribution of the mean theta phase offset across recording sessions. The mean theta phase offset was obtained by averaging across neurons recorded during the same session, sorted by recording depth (L2-4: 250-500µm; L5: 500-700µm; L6: 700-950µm). *Arrow*: median of the theta phase offset across sessions. The mean phase offset was not significantly different between deep and superficial layers across sessions (L6 vs. L5: $p = 0.55$; L6 vs. L2-4: $p = 0.43$).

R. Distribution of the amplitude of the spatial shift across theta phases for CA1 (*left*) or V1 (*right*) neurons whose responses were significantly shifted. The amplitude of the spatial shift was measured from the amplitude of the sinusoid that best fitted the maximum curve of the spatial cross-correlogram (*black curve* in **L**).

S. Ahead vs. behind shifts in the response profile of each neuron across theta phase (extrema of the black curve in **K**). In CA1 neurons (*left*), the shift across theta phase tended to be larger ahead than behind (p = 0.039, rank sum test). In V1 neurons (*right*), this shift was similar ahead and behind (p = 0.77, rank sum test)

T. Pearson's correlation across theta cycles between V1 and CA1 theta modulation of decoding errors (*x-axis*), compared to the same quantity measured after shuffling time points within position and speed bins (*y-axis*). The theta modulation of decoding errors was measured from the absolute amplitude of the best fitting sinusoid on every theta cycle. Each dot corresponds to one recording session (n = 27). The correlation was higher for the actual data than for the shuffle control ($p = 3.10^{-5}$ Student's t-test) and significant in 63% of the sessions (17/27, *filled circles*).

Supplemental References

S1. Bartho, P., Hirase, H., Monconduit, L., Zugaro, M., Harris, K.D., and Buzsaki, G. (2004). Characterization of Neocortical Principal Cells and Interneurons by Network Interactions and Extracellular Features. J. Neurophysiol. *92*, 600–608.

S2. Sirota, A., Montgomery, S., Fujisawa, S., Isomura, Y., Zugaro, M., and Buzsaki, G. (2008). Entrainment of Neocortical Neurons and Gamma Oscillations by the Hippocampal Theta Rhythm. Neuron, 683–697.

S3. Senzai, Y., Fernandez-Ruiz, A., and Buzsáki, G. (2019). Layer-Specific Physiological Features and Interlaminar Interactions in the Primary Visual Cortex of the Mouse. Neuron *101*, 500-513.e5.
